# CD8+ T Lymphocyte Epitopes From The Herpes Simplex Virus Type 2 ICP27, VP22 and VP13/14 Proteins To Facilitate Vaccine Design And Characterization

**DOI:** 10.3390/cells2010019

**Published:** 2013-01-04

**Authors:** Rebecca J. Platt, Tansi Khodai, Tim J. Townend, Helen H. Bright, Paul Cockle, Luis Perez-Tosar, Rob Webster, Brian Champion, Timothy P. Hickling, Fareed Mirza

**Affiliations:** 1 Biotherapeutics and Translational Research, Pharmacokinetics, Dynamics & Metabolism, Pfizer Global Research and Development, Sandwich, Kent, CT13-9NJ, UK; E-Mails: Rebecca.Platt@Covance.com (R.J.P.); tim.j.townend@gsk.com (T.J.T.); Luis.Perez@ablynx.com (L.P-T.); rob.webster@pfizer.com (R.W.); fareed.mirza@novartis.com (F.M.); 2 New Opportunities Unit, Pfizer Global Research and Development, Sandwich, Kent, CT13 -9NJ, UK; E-Mails: tansi.khodai@googlemail.com (T.K.); h.h.bright@btinternet.com (H.H.B.); 3 Vaccine Research Unit, Pfizer Global Research and Development, Sandwich, Kent, CT13-9NJ, UK; E-Mails: paul.cockle@pfizer.com (P.C.); brian.champion@pfizer.com (B.C.)

**Keywords:** HSV-2, CD8+ Epitope, Vaccine, Infection

## Abstract

CD8+ T cells have the potential to control HSV-2 infection. However, limited information has been available on CD8+ T cell epitopes or the functionality of antigen specific T cells during infection or following immunization with experimental vaccines. Peptide panels from HSV-2 proteins ICP27, VP22 and VP13/14 were selected from *in silico* predictions of binding to human HLA-A*0201 and mouse H-2Kd, Ld and Dd molecules. Nine previously uncharacterized CD8+ T cell epitopes were identified from HSV-2 infected BALB/c mice. HSV-2 specific peptide sequences stabilized HLA-A*02 surface expression with intermediate or high affinity binding. Peptide specific CD8+ human T cell lines from peripheral blood lymphocytes were generated from a HLA-A*02+ donor. High frequencies of peptide specific CD8+ T cell responses were elicited in mice by DNA vaccination with ICP27, VP22 and VP13/14, as demonstrated by CD107a mobilization. Vaccine driven T cell responses displayed a more focused immune response than those induced by viral infection. Furthermore, vaccination with ICP27 reduced viral shedding and reduced the clinical impact of disease. In conclusion, this study describes novel HSV-2 epitopes eliciting strong CD8+ T cell responses that may facilitate epitope based vaccine design and aid immunomonitoring of antigen specific T cell frequencies in preclinical and clinical settings.

## 1. Introduction

Herpes Simplex Virus Type 2 (HSV-2) is responsible for recurrent genital ulcers, cold sores, herpetic stromal keratitis and fatal encephalitis, and contributes to morbidity seen in immunocompromised individuals and neonates with severe infection. HSV-2 is a major global health concern with an estimated 123 million people infected in Sub-Saharan Africa (15–49 years) compared with 29.8 million in the US and 20.9 million in Europe [1,2]. Long term treatment of HSV-2 infection with anti-viral drugs has been successful in managing the disease by reducing recurrent activation, limiting viral shedding and reducing transmission of the virus. Although therapeutic treatments are widely available, development of a prophylactic vaccine to achieve sterilizing immunity is highly desirable. Two clinical vaccine studies aimed at generating neutralizing antibodies and CD4+ T cell responses have resulted in limited success. The former prophylactic vaccine delivered HSV-2 glycoproteins (gD and gB) in combination with the adjuvant MF59 and generated neutralizing antibodies but demonstrated transient efficacy in only a minor cohort of individuals [3,4]. Another prophylactic approach used the HSV-2 glycoprotein-D-subunit vaccine with alum and 3-O-deacylated-monophosphoryl lipid A (MPL) though efficacy was only observed in HSV-1 and HSV-2 seronegative females [5,6]. 

Vaccine strategies aimed at generating CD8+ T cell responses and targeting additional immunogenic HSV-2 proteins have potential to result in better efficacy [7,8,9]. Increased knowledge of CD8+ T cell epitope sequences will aid vaccine design by the incorporation of immunogenic peptide sequences into vaccines and allow detailed phenotypic analysis and characterization of the kinetics of the cellular immune response after immunization. The underlying mechanistic T cell control of HSV-2 is not well understood, however emerging data has highlighted the importance of CD8+ T cell control in HSV-2 reactivation and for maintaining but not establishing latency [10,11]. Zhu *et al.* have shown that CD8+ T cells have a key role in the control and progression of HSV-2 by localizing at the site of infection [12]. Adoptive transfer experiments using OVA specific CD8+ T cells in HSV-2 Tk-OVA infected mice resulted in clearance of infection that could be reversed by the *in vivo* neutralization of IFN-γ [13]. Interestingly, the protein ICP4 has recently been shown to be the target of granzyme B mediated degradation, which results in non lethal viral inactivation [14]. CD8+ T cells were shown to control viral reactivation in cultured trigeminal ganglia (TG) from HSV-2 infected mice. Depleting CD8+ T cells from cultures promoted increased rates of HSV-2 reactivation [15]. Clinical studies evaluating the diversity of CD8+ restricted T cell responses to HSV-2 proteins have shown that the greatest frequency of responses are directed against tegument proteins but are only found in 50% of patients [16] and that CD4+ responses are broader than CD8+ [17]. The CD8+, IFN-γ+ T cell responses were restricted to the VP22, ICP0 and ICP4 antigens with no additional responses detected *ex vivo* for ICP27, ICP22 or gD proteins. These important findings indicate that tegument protein specific T cells may play an important role in controlling HSV-2 infection and provide attractive opportunities for immune intervention. 

ICP27, VP22 and VP13/14 have previously been shown to elicit strong T cell responses in mice and have been used for exploratory vaccine studies [18,19,20,21]. With limited epitope information available on ICP27, VP22 and VP13/14 we sought to identify epitope sequences in a preclinical BALB/c mouse model using a combination of computational prediction, ELISpot and flow cytometry methods. Responses to VP22 and VP13/14 have also been demonstrated in humans [22]. Translational studies using HLA-A*02-peptide binding assays and human T cell lines generated by peptide driven expansions from well conserved ICP27, VP22, and VP13/14 epitope sequences exhibited functional activity in response to their cognate peptide stimulation *in vitro*. In order to induce CD8+ T cell responses to HSV-2 proteins through active immunization, we constructed DNA vaccines encoding full length ICP27, VP22, and VP13/14 proteins. To facilitate strong MHC class I mediated expression of viral proteins, DNA was delivered using the proprietary gold particle mediated epidermal delivery (PMED^TM^) with the ND10 delivery device [23]. CD8+ T cell responses were evaluated using ELISpot and mobilization of CD107a after peptide stimulation. Epitope specific T cell responses generated from PMED DNA immunization showed a narrowed and more focused T cell response towards ICP27, VP22 and VP13/14 as compared with HSV-2 infection in BALB/c mice. These T cell epitopes may facilitate vaccine design and aid immunomonitoring of peptide specific T cell responses in pre-clinical and clinical studies.

## 2. Results and Discussion

### 2.1. Identification of Epitopes in ICP27, VP22 and VP13/14

Computer aided epitope identification has proven to be a very powerful tool to select specific areas within large protein sequences with immunogenic potential. To aid the identification of CD8+ restricted T cell epitopes we used linear based epitope detection methods, including ELISpot, to narrow 15mer peptide specific responses to 9mer peptides for *in vitro* T cell stimulation experiments using BALB/c mice infected with HSV-2. The peptides derived from ICP27, VP22 and VP13/14 were identified based on the ability to bind Kd, Dd, Ld and HLA-A*02 MHC molecules using the Immune Epitope Database [24] (Table 1). The sequence from ICP27_(318-326) _has previously been shown to elicit strong T cell responses in preclinical experiments and was included as a positive control [18]. Additionally the Flu matrix M1_(58-66)_ epitope served as positive control for the bioinformatics screening and for T2 stabilization studies.

**Table 1:** Sequence information and predicted IC50 (nM) binding values for ICP27, VP22 and VP13/14 selected peptides.

Epitope	Peptide Sequence	Predicted ^a^ IC50 (nM) for Kd	Predicted^ a^ IC50 (nM) for Dd	Predicted^ a^ IC50 (nM) for Ld	Predicted ^a^ IC50 (nM) for HLA-A2
ICP27(314-322)	TLVAHGPSL	29704.2	29811.3	29903.8	172.1
ICP27(318-326) *	HGPSLYRTF *	29523.2	41.4	26435.4	24491.7
ICP27(319-327)	GPSLYRTFA	37218.7	32191.1	36928.3	24083.7
ICP27(424-432)	DIASFVLVI	32248.6	16323.4	44299.6	12567.5
ICP27(425-433)	IASFVLVIL	27247.8	29428.1	38689.5	5961
ICP27(427-435)	SFVLVILAR	31071.1	33613.3	45487.2	23983
ICP27(428-436)	FVLVILARL	15291.9	31066.4	39404.5	44.2
ICP27(459-467)	TMHFYIPGA	39602.4	29117.4	44287.4	37.3
ICP27(462-470)	FYIPGACMA	2073.3	30525.3	43726.8	13482.9
ICP27(464-472)	IPGACMAGL	22701.7	8136.7	322.7	21245.3
ICP27(479-487)	HRQECSSRV	32214.5	31069.2	39806.7	19930.4
ICP27(482-490)	ECSSRVCEL	31674.4	23031	43090.6	22306.7
ICP27(484-492)	SSRVCELTA	37431.5	29921.3	44401.6	23541.2
ICP27(499-507)	LYVHGKYFY	31170.9	31676	42144.7	24253.4
ICP27(500-508)	YVHGKYFYC	39488.8	31902.1	31410.8	4141.3
ICP27(502-510)	HGKYFYCNS	39256	30676.7	44620	24494
ICP27(504-512)	KYFYCNSLF	1731.1	19533.6	35494.7	22076.5
VP22(46-54)	MRARPRGEV	33458	31986.6	40180.1	22734.7
VP22(49-57)	RPRGEVRFL	35468.7	32591.2	15593.8	24194.2
VP22(144-152)	PAQADSAVL	31273.4	27008	39887.1	22567.6
VP22(146-154)	AQADSAVLL	28650.2	29610.5	39773.3	219.8
VP22(170-178)	QGLAKKLHF	36005.8	25105.8	28660.9	24422.6
VP22(171-179)	GLAKKLHFS	38907.3	30342.7	44645.9	3066.5
VP22(172-180)	LAKKLHFST	36466.3	32481.7	41828.9	22976.2
VP22(173-181)	AKKLHFSTA	36250.5	33000.5	44582.4	24606.2
VP22(197-205)	NKRVFCAAV	30008.2	31775.2	44919.6	22655.2
VP22(200-208)	VFCAAVGRL	1306.3	32999.6	9018.9	21578.2
VP13/14(224-232)	FYPCPDSAF	16119.3	1274.8	39803	24361.5
VP13/14(225-233)	YPCPDSAFG	39192.9	34274.1	34907.2	24514.4
VP13/14(228-236)	PDSAFGLSR	39770.5	33330.2	45411.5	24833.9
VP13/14(229-237)	DSAFGLSRV	31063.7	29721.2	44388.9	21332.2
VP13/14(242-250)	FASPADPKV	36540	26313.6	7025.5	743
VP13/14(243-251)	ASPADPKVF	37288.4	6838.7	31207.7	24557.9
VP13/14(244-252)	SPADPKVFF	37123.2	13334.2	232.3	24985.9
VP13/14(394-402)	TYIATGALL	21.5	25985.1	34758.1	19830.3
VP13/14(395-403)	YIATGALLA	37750	31700.8	44153.7	162.8
VP13/14(414-422)	LPREAAFAG	39383.4	33334.8	26281.3	24771.1
VP13/14(415-423)	PREAAFAGR	39228.2	32630.7	43207.1	24858.4
VP13/14(419-427)	AFAGRVLDV	25977.5	20910.7	40665.1	11777.1
VP13/14(421-429)	AGRMTYIAT	36221.7	30484.7	44486.5	24623
VP13/14(589-597)	TQPLYARTT	39452.7	25084.5	45112	22116.8
VP13/14(590-598)	QPLYARTTP	28489.2	31596.9	30047.2	24585.7
VP13/14(591-599)	PLYARTTPA	28610.6	28076.8	44528.9	2426.4
VP13/14(592-600)	LYARTTPAK	23103.7	30223.7	45027.4	24767.2

* Denotes a previously described epitope in BALB/c mice (see reference [18]);^ a^ Predictions were performed using IEDB www.immuneepitope.org (see reference [24] and methods);^ b ^Known HLA-A2 binder.

### 2.2. Identification of Epitopes in ICP27, VP22 and VP13/14 from HSV-2 Infected BALB/c Mice

**Figure 1:**
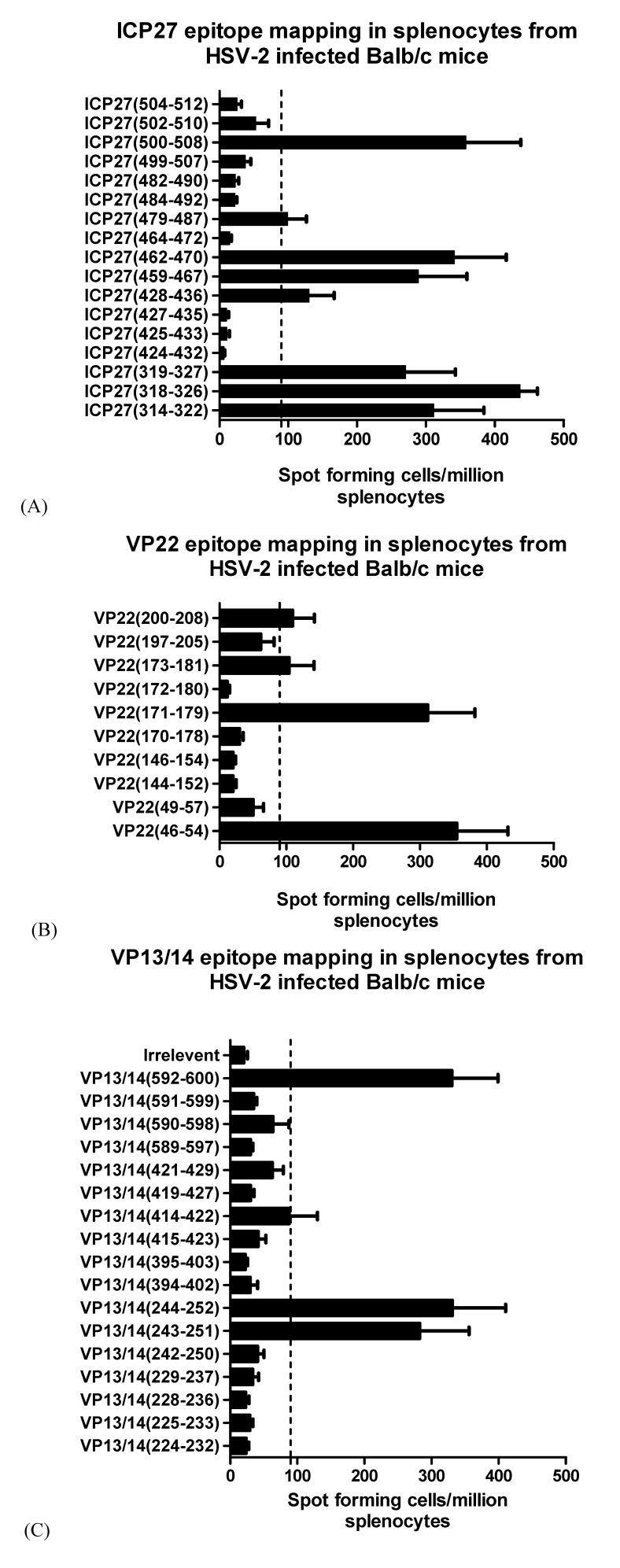
Identification of epitopes in ICP27, VP22 and VP13/14 from HSV-2 infected BALB/c mice. **B**ALB/c mice were infected with 1 × 10^3^ pfu of HSV-2 (strain 333) through intra-vaginal administration. After 14 days splenocytes were obtained and pulsed individually (5 × 10^5^ cells) with 10 µg/mL of (A) ICP27, (B) VP22 and (C) VP13/14 derived peptides in an overnight IFN-γ ELISPpot assay. Counts are presented as spot forming cells per million splenocytes with standard error of means (SEM). Cut off values 2 SD over background were considered significant and spots reaching 90 SFU or above were defined as positive.

HSV-2 infection studies in BALB/c mice were performed to identify strong peptide specific T cell responses. BALB/c mice were infected with HSV-2 and monitored for 14 days before spleens were removed and harvested for splenocytes. IFN-γ ELISpots were used to measure T cell responses after overnight stimulation with peptide. Intra-vaginal infection of mice with HSV-2 strain 333 resulted in a broad detectable cellular immune response towards ICP27, VP22 and VP13/14 (Figure 1 A,B,C and Table 2). The previously reported mouse epitope ICP27_(318-326) _elicited a strong response after infection (n = 4) as expected. Peptide sequences that have not been previously described for ICP27_(500-508),_ ICP27_(459-467), _ICP27_(479-486)_, and ICP27_(428-436)_ were also positive for IFN-γ T cell responses (≥90SFC/million splenocytes) after peptide stimulation. Interestingly, overlapping amino acid sequences from ICP27_(314-322)_ and ICP27_(319-327)_ gave lower responses than ICP27_(318-326)_ (Figure 1A). Strong peptide specific responses were seen with ICP27_(462-470)_ and ICP27_(500-508) _which were comparable to the published sequence ICP27_(318-326)_ (Figure 1A). Further epitope screening of VP22 and VP13/14 peptides resulted in three strongly positive epitopes being identified (VP22_(46-54),_ VP22_(171-179)_ and VP22_(200-208)_) and two from VP13/14 (VP13/14_(244-252) _and VP13/14_(592-600)_) shown in Figure 1B and Figure 1C. Splenocytes pulsed with individual peptides from naive mice (n = 2) gave background responses below 16.5 SFC per million splenocytes (Table 2). Although identification of potential epitopes in mice is highly informative, further work is required to assess the involvement of T cell subsets that are able to induce IFN-γ production. We have made use of peptides that span 9 amino acids which have a natural affinity for MHC class I molecules, however it has been shown that the MHC class II molecule requires a 9mer sequence to obtain core binding [25,26]. To exclude the possibility of CD4+ mediated IFN-γ responses after infection with HSV-2, cells sorted for CD4+ and CD8+ T cells may be used in peptide pulsing studies. Additionally, measurement of total IFN-γ positive responses to whole virus, protein or a full set of overlapping peptides spanning the full length of viral antigens would allow ranking of the magnitude of peptide specific responses. From the predicted IC50 binding values it is apparent which BALB/c allele each peptide is restricted to, however experimental validation using cell lines expressing H2 molecules are required for detailed pre-clinical MHC-peptide tetrameric and phenotypic analysis of peptide specific T cells are required. The identification of epitopes in HSV-2 viral proteins were identified in an infection setting with a virus presenting multiple H2 restricted T cell epitopes from different proteins. To translate our findings with clinical significance it is necessary to determine human HLA-A*02 restricted epitopes in HSV-2 proteins.

**Table 2:** Summary of epitopes identified from HSV-2 proteins in BALB/c mice after infection and immunization with ICP27, VP22 and VP13/14 DNA vaccines.

BALB/c Epitope	Peptide Sequence	IFN-γ Responses (SFC) after HSV-2 Infection in BALB/c mice (n = 4)	IFN-γ Responses (SFC) in Mice Immunized with Full Length DNA Vaccine (n = 6) ^ψ^	IFN-γ Responses (SFC) in Uninfected BALB/c Mice (n = 2)
ICP27(318-326) *	HGPSLYRTF	436.2	304.4	16.5
ICP27(428-436)	FVLVILARL	129.7	16.9	7.5
ICP27(459-467)	TMHFYIPGA	288.5	71	5.5
ICP27(479-487)	HRQECSSRV	98.75	12.5	5.5
ICP27(500-508)	YVHGKYFYC	357.7	247	4.5
VP22(46-54)	MRARPRGEV	354.7	4.5	3
VP22(171-179)	GLAKKLHFS	311.2	23.8	8
VP22(200-208)	VFCAAVGRL	109.2	189.6	9.5
VP13/14(244-252)	SPADPKVFF	330.7	ND **	4.5
VP13/14(592-600)	LYARTTPAK	330	ND **	8.5

* Denotes a previously described epitope in BALB/c mice (see reference [18]);^ ψ ^Mice immunized with DNA vaccine encoding ICP27, VP22 or VP13/14 using PMED device. Naïve mice immunized with DNA encoding mock antigen showed mean background responses for individual peptides less than 5 SFU/million splenocytes (n = 6)’ ** ND (Not determined).

### 2.3. Determination of HLA-A*02 Restricted HSV-2 Epitopes

**Figure 2:**
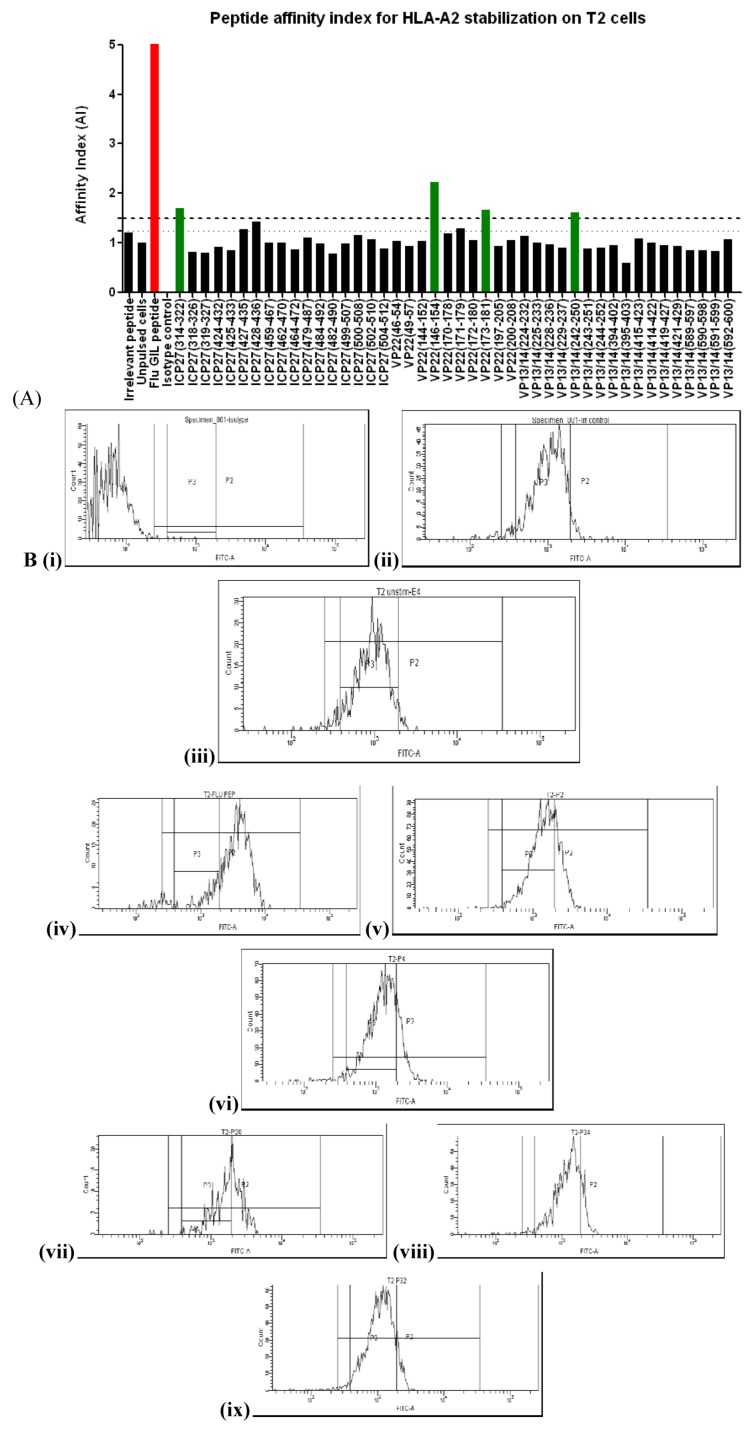
HLA-A2 stabilization by exogenously loaded peptides on T2 cells. **T**2 cells were plated out in a 96 well plate. Peptide (50 µg/mL) was added and the cells were left to incubate overnight. (A) Stabilized expression of HLA-A2 was detected using a FITC conjugated BB7.2 antibody. The HLA-A2 restricted Flu matrix M1_(58-66)_ (GILGFVFTL) peptide was used as positive control at the same concentration as the HSV-2 test peptides (shown in red). (B) Relative peptide binding affinity was calculated from the formula: AI = (MFI_1_– MFI_0_)/(MFI_2_ – MFI_0_) (see methods). The dashed line represents an AI of ≤1.5 (high affinity) and the dotted line represents an AI of ≥1.25 (intermediate affinity). (i) FITC Isotype control, (ii) Irrelevant control, (iii) Unpulsed cells, (iv) Influenza matrix _(58-66)_, (v) ICP27_(314-322)_, (vi) ICP27_(428-436)_, (vii) VP22 _(146-154)_, (viii) VP22 _(173-181)_, (ix) VP13/14 _(242-252)_

Epitope predictions highlighted the potential for peptide binding to the MHC class 1 molecule with high affinity (affinity index ≥1.5) in comparison to the Flu Matrix M1 epitope [27]. T2 cells were used to determine the affinity index of peptides able to bind and stabilize HLA-A*02 MHC molecules. Figure 2A shows the AI for all 44 peptides calculated from mean fluorescent intensities. Four peptide sequences, ICP27_(314-322)_ ,VP22_(146-154)_ ,VP22_(173-181)_ , VP13/14_(242-250)_ were able to reach an AI > 1.5 (high affinity) and ICP27(428-436) scored 1.42 (intermediate affinity) (Figure 2A). Figure 2B shows shift in intensity of HLA-A*02 on T2 cells for the strongest ICP27, VP22 and VP13/14 peptide binders. The human donors had no history of HSV-2 infection, but are likely to have been exposed to HSV-1. Alignment of the HLA-A*02 restricted peptides identified for HSV-2 with the sequences for HSV-1 ICP27, VP22 and VP13/14 proteins showed close homology for three out of four peptides. Although samples from our donors required repetitive *in vitro* re-stimulation in order to identify specific responses, there have been reports demonstrating HSV-2 specific T cells in seronegative individuals [28] which is similar to T cells found in HIV-1 exposed but uninfected individuals [29,30,31]. 

**Figure 3:**
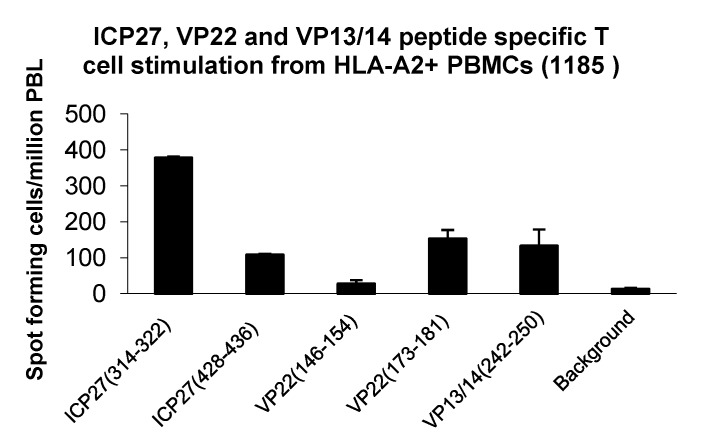
Generation and expansion of HSV-2 peptide specific T cells from an HLA-A*02+ donor (1185): PBL were derived from an HLA-A*02+ donor and stimulated with peptides from ICP27_(314-322),_ ICP27_(428-436),_ VP22_(146-154),_ VP22_(173-181)_ and VP13/14_(242-150)_. After 4 stimulations cells were assessed for peptide specific responses by an overnight IFN-γ ELISpot assay. This figure represents the original count data from the ELISpot plate with standard deviations (SD).

With HSV-1 sequence conservation between 66.7% and 100% (6/9 to 9/9 amino acid identity, shown in Table 3) and reports describing T cell responses in HSV-2 seronegative individuals [29], we screened HLA-A*02+ healthy donors and established peptide derived T cell lines from repetitive peptide pulsing from donor 1185 (Figure 3). We were able to identify peptide specific T cell responses (background +2 SD) from ICP27_(314-322),_ ICP27_(428-436)_, VP22_(173-181)_ and VP13/14_(242-250)_ (Figure 3). This finding indicates a presence of a small frequency of functionally reactive peptide specific T cells able to respond to peptide stimulation. *Ex vivo* screening and phenotyping of T cells derived from both healthy donors and HSV-2 positive patients using peptide-MHC tetrameric complexes may provide additional information on frequency and functionality of HSV-2 specific T cells. Table 3 summarizes the HLA-A2 epitopes identified in this study. 

**Table 3:** Summary of epitopes identified from HSV-2 proteins in a healthy human HLA-A2+ donor (1185) with % sequence homology to HSV-1. The set threshold for high affinity binding peptides was given an AI of ≥1.5, and for intermediate affinity peptides was given an AI of 1.25–1.5.

HLA-A2 Restricted Epitope	Peptide Sequence	% SEQUENCE Homology to HSV-1 ^a^	Affinity Index (AI)^ b^	Mean SFU/million PBL
ICP27(314-322)	TLVAHGPSL	100	1.70	377.5
ICP27(428-436)	FVLVILARL	88.8%	1.42	107.5
VP22(173-181)	AKKLHFSTA	88.8%	1.28	152.5
VP13/14(242-250)	FASPADPKV	66.7%	1.61	132.5

^a ^Alignments were performed using genebank HSV-1 ICP27 accession AB235845.1, HSV-1 VP22 accession NP_044651, HSV-1 VP13/14 accession NP_044649. HSV-2 alignments were performed with ICP27 accession NP_044525, VP22 accession NP_044519, VP13/14 accession ABS84906; ^b ^Assessed by T2 stabilization. Affinity index > 1.5 high affinity, 1.25-1.49 was considered as an intermediate affinity peptide.

During the course of the present study, two papers were published that described peptide selection, pre-clinical efficacy [32] and Phase I clinical trial [33] data for a polyvalent anti-HSV-2 vaccine consisting of 32 peptides from HSV-2 proteins. These studies included a single 35 amino acid peptide from ICP27 (amino acids 410-444), but did not include any peptides from VP22 or VP13/14. The aim of those studies was to generate both CD4+ and CD8+ cellular immunity. We have described a peptide from the same region of ICP27 (amino acids 428-436) with an intermediate affinity for HLA-A2 that induced reactive CD8+ T cells in natural infection (Table 3) and also described a further peptide from ICP27 (314-322) that had a higher affinity for HLA-A2 (Figure 2A), and a stronger response in our donor (Table 3). It is not apparent from the published preclinical study what impact the ICP27 peptide had in the vaccine, nor is it possible to say whether the alternative peptide we identified would improve efficacy. However, it is important for future vaccine design to be able to identify the best candidate peptides for driving strong immune responses that can provide clinical efficacy. 

### 2.4. HSV-2 DNA Vaccines Delivered by PMED Generate High Frequencies of CD8+ Peptide Specific T Cells

**Figure 4:**
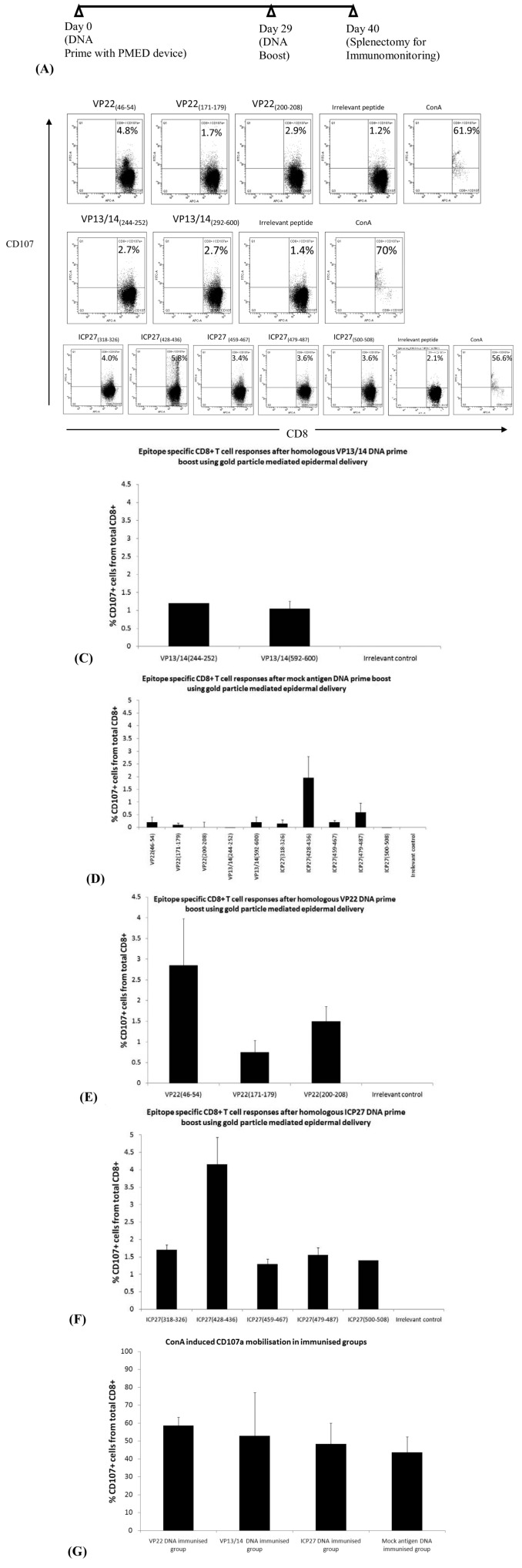
Mobilization of CD107a on peptide specific CD8+ T cells after DNA vaccine administration encoding the HSV-2 ICP27, VP22 and VP13/14 antigens. (A) BALB/c mice (n = 6) were immunized using the DNA prime-boost regimen using particle mediated epidermal delivery with full length monovalent vaccines (ICP27, VP22 and VP13/14). On day 40 splenocytes were harvested, pooled in their respective groups and pulsed with peptide for 4 hours in the presence of anti-CD107a-FITC antibody. (B) FACS plots are shown as CD107a+/CD8+ T cells as a percentage of total CD8+ T cells for VP22, VP13/14 and ICP27 peptide specific response. Irrelevant peptide control and ConA CD107a+/CD8+ responses are shown as negative and positive controls for CD107a degranulation. (C, D, E, F) Percentage of CD107a+/CD8+ T cell responses shown with irrelevant peptide responses subtracted from the total CD107a+/CD8+ T cell percentages for each of the HSV-2 DNA immunization group. (E) Graph showing CD107a+/CD8+ T cell responses in mice immunized with a mock antigen and pulsed with ICP27, VP22 and VP13/14 peptides. (G) Graph showing CD107a+, CD8+ T cell degranulation after ConA stimulation.

Full length DNA vaccines encoding codon optimized ICP27, VP22 and VP13/14 sequences were constructed for vaccine delivery using the PMED device. DNA vaccine delivery using the PMED gene gun has demonstrated success in the clinic and provides a safe and alternative approach to needle based intramuscular delivery requiring milligrams of DNA [34,35,36]. Gold particles containing 2 µg of DNA were given as a single actuation to the shaved abdomen of BALB/c mice without adjuvant. Mice were then given a second booster vaccine 29 days after the primary immunization. Spleens were removed on day 40 to assess T cell responses using IFN-γ ELISpot and mobilization of CD107a after peptide stimulation (Figure 4 A-G and Table 2). Table 2 shows the peptide specific IFN-γ ELISpot responses after DNA vaccination to each HSV-2 protein. Strong ICP27 specific responses were observed to the previously unidentified ICP27_(500-508) _epitope (247 SFC/million PBL), with the strongest response restricted to the ICP27_(318-326) _epitope (304 SFC/million PBL). Interestingly no other T cell responses were seen to epitopes that elicited ICP27 specific T cells after HSV-2 infection. This finding suggests that vaccine driven responses to ICP27 generated T cells to immunodominant sequences. CD107a mobilization shows broad degranulation of ICP27 peptide specific CD8+ T cells with frequencies in excess of background levels (Figure 4B, E and F). Interestingly, IFN-γ positive responses were only seen for the VP22_ (200-208)_ epitope after VP22 DNA immunization. In contrast, 3.6% and 1.7% (including irrelevant peptide background subtraction) of total CD8+ T cells degranulated after VP22_(46-54) _and VP22_(200-208) _after peptide stimulation respectively (Figure 4B and C). Collectively, this data illustrates potential immunodominance and broad functionality of cytokine secreting CD8+ T cells raised after DNA immunization. Similarly like ICP27, strong IFN-γ T cell responses were observed to the VP22 epitopes, VP22(46-54), VP22(171-179) and VP22(200-208) after infection with HSV-2. CD8+ T cells degranulating in response to VP13/14_(244-252)_ and VP13/14_(592-600)_ peptide stimulation after VP13/14 DNA immunization were present at a modest frequency of 1.3% of total CD8+ T cells. 

### 2.5 Potential Role of ICP27 in Protection Against HSV Challenge Following Immunization

**Figure 5:**
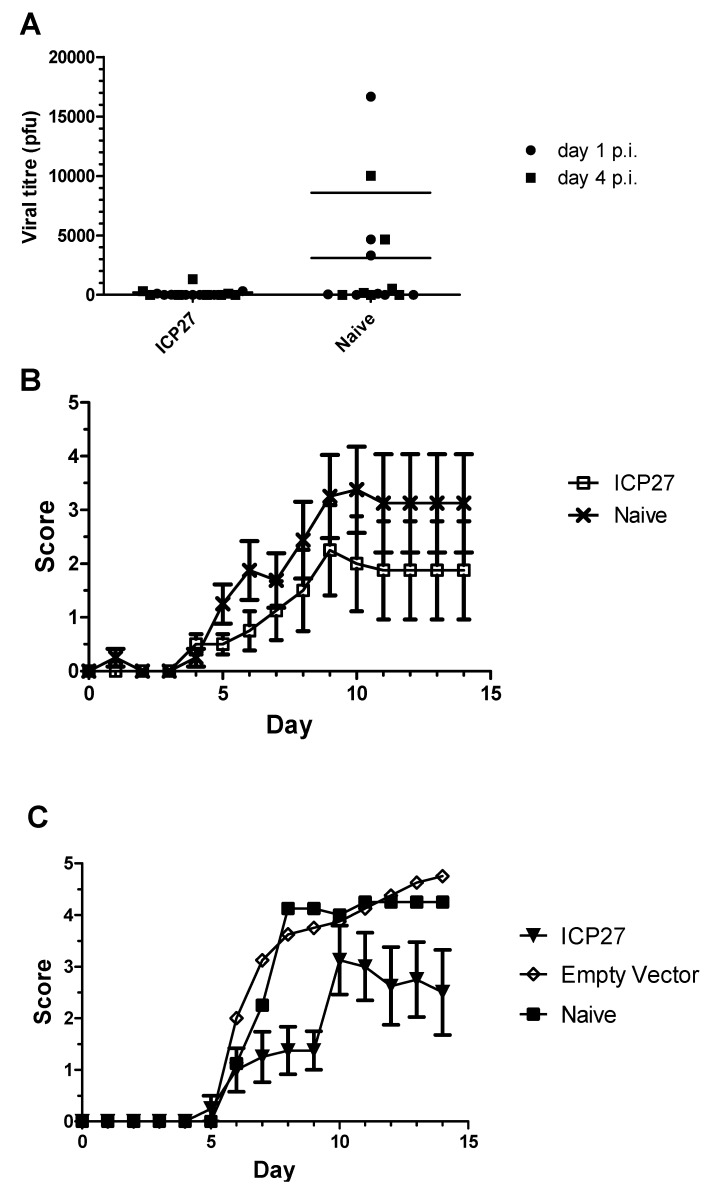
*A role for ICP27 in protection against HSV challenge following immunization.* A DNA vaccine against ICP27 was used to immunize mice prior to challenge with HSV-2 in intravaginal (A, B) and intranasal (C) models of infection. A) Virus shedding was detected by viral plaque assays at days 1 (closed circles) and 4 (closed squares) post infection. B) Mean clinical score for ICP27 vaccinated (open squares) and naive (crosses) groups at each day for fourteen days post-infection. C) Mean clinical scores for ICP27 vaccinated (closed triangle), mock vaccinated (open rhombus) and naive (closed squares) groups at each day for fourteen days post-infection.

Specific cellular responses to ICP27 were generated both by infection and by DNA vaccination. Vaccinated mice were challenged with HSV-2 to investigate the potential of these responses to protect against infection in both intravaginal and intranasal challenge models. Vaccination of mice with ICP27 provided protection against viral shedding in the intravaginal model, with five out of eight animals shedding no virus at either one or four days post infection compared with three out of eight controls (Figure 5A). Mean viral titers were 225 and 8590 for vaccinated and unvaccinated groups at day four post-infection, respectively. Vaccinated mice demonstrated a trend towards lower clinical scores than unvaccinated counterparts, although this was not demonstrably significant (Figure 5B). Vaccination with ICP27 was more protective in the intranasal challenge model, with vaccinated mice showing reduced clinical scores and a delayed onset of more significant disease (Figure 5C). 

T cell control in establishing latency in HSV-2 infection is not fully understood. Additional work using T cell lines and clones sorted from HSV-2 patients in transwell and migration experiments may allow better understanding of therapeutic approaches to control or eliminate HSV-2 from infected cells. CD8+ T cell recruitment to sites of infection has recently been shown to depend heavily upon CXCR3+ CD4+ T cell induced mobilization through the CXCL9 and CXCL10 gradient [37]. This important finding suggests that vaccines should be designed to induce both CD8+ T cells with CD4+ T cell help. Live imaging techniques using antigen specific T cell adoptive transfer experiments in RAG knockout mice would provide valuable data on localization of T cells and subsequent mode of action. 

It has been shown for vaccinia virus that the route of infection may skew peptide specific responses to certain epitopes or enhance and diminish dominant CD8+ T cell responses [38,39,40]. In this study, we have not assessed intra-ocular or other routes of infection. Furthermore, rational single amino acid modifications to peptides known to elicit a stimulatory response may enhance immunogenicity and therefore result in a more favorable outcome in an epitope based vaccine and disease challenge setting [41]. ICP27, VP22 and VP13/14 DNA vaccines delivered using the PMED ND10 device elicited strong CD107a+/CD8+ T cells after peptide stimulation. It is interesting to note that the peptide specific T cell frequencies could not be compared between T cells positive for CD107a expression and IFN-γ ELISpot after peptide stimulation. Furthermore, BALB/c mice infected with HSV-2 showed a greater frequency of T cells specific for ICP27 and VP22 epitopes than mice immunized with DNA vaccines (Table 2). It is important to note that mice infected with HSV-2 also displayed a greater coverage of epitope specific responses than with DNA vaccination which skewed ICP27 and VP22 specific responses to ICP27_(318-326),_ ICP27_(500-508)_ and VP22_(200-208)_ epitopes. Due to the polyfunctional nature of T cells generated through vaccination, further work is required to dissect the proportion of HSV-2 pentamer/tetramer specific CD8+/CD107a+ cells capable of mounting IFN-γ+, IL-2+ and TNF-α+ responses. Further translational experiments using HLA-A*02 restricted epitopes described in this study on HLA-A*02+ infected transgenic mice or HSV-2 seropositive donors may provide a clearer understanding of the frequency and functionality from ICP27, VP22 and VP13/14 restricted T cells *ex vivo*. Additionally for vaccine development, translational experiments using *in vitro* DC:T cell co-culture experiments would provide a clearer understanding of the immunogenicity and functionality of HLA restricted HSV-2 epitopes. 

## 3. Experimental Section

### 3.1. Peptide Selection for HSV-2 ICP27, VP22 and VP13/13 Epitopes

An initial screening of responses to peptides was performed with 15mer peptides overlapping by 10 amino acids derived from ICP27 (Accession number, NP_044525, version GI: 9629325), VP22 (NP_044519, GI: 9629319) and VP13/14 (ABS84906, GI: 154744659) (Data not shown). Peptides were synthesized by Sigma-Aldrich Corp, St. Louis, MO. Sequences that were found to give positive responses were then selected for subsequent fine mapping and characterization. Peptide selection was restricted to peptides spanning 9 amino acids and was performed on mouse MHC class I molecules for H2-Dd, H2-Kd, H2-Ld and human HLA-A*0201 using the Immune Epitope Database (IEDB) [24]. In total, 44 peptides were selected with theoretical IC50 values ranging from 21.5 to 45487.2 nM spanning selected regions of ICP27, VP22 and VP13/14 proteins, as shown in Table 1. Overlapping amino acid sequences of differing IC50 values spanning 15-mer sequences were included to assess the impact of residue modifications on the extent of immunogenicity.

### 3.2. Peptide Synthesis

Peptides of 9 amino acids in length were synthesized at >85 % purity for epitope mapping (Sigma–Aldrich). In total 44 peptides were obtained and dissolved in 100% DMSO (Sigma) at a stock concentration of 10 mg/mL. Peptides were used at a final concentration of 50 µg/mL unless otherwise stated. For peptide stabilization studies the well defined HLA-A*0201 sequence from Influenza Matrix Protein M1 GILGFVFTL_(58 - 66)_ was used as a positive control (Anaspec, CA, US) [42].

### 3.3. Animals

Six to eight week old female BALB/c mice (Charles River, UK) were used in the studies. All experiments were carried out in compliance with the UK legislation and subject to local ethical review. Humane endpoints were strictly adhered to.

### 3.4. Intravaginal HSV-2 Challenge Model

Six days prior to infection, mice were given 20 µg of Depo Provera (Medroxyprogesterone) (Pfizer, Ltd) subcutaneously to ensure mice enter diestrus when infected. On day 0, mice (n = 10) were infected with 1 × 10^3^ pfu of HSV-2 strain 333 in a total volume of 20 µL PBS by intravaginal administration. The control group (n = 2) were given PBS by the intravaginal route. On days 1 and 4 post-infection, vaginal lavage was collected by pipeting 50 µL of phosphate buffered saline in and out of the vagina several times, transferred to vials and stored at −80 °C until assayed for viral load using plaque assay. Mice were monitored for 14 days and their health was checked daily using a clinical score system and by recording body weight. Pathology was assessed using a 5-point score as follows: Healthy – no apparent infection = 0, Slight redness = 1, Redness and swelling = 2, Severe redness of surrounding area = 3, Ulceration and/or hair-loss = 4, score of 4 in addition to 2 additional clinical signs = 5. All procedures were carried out under the authority of a UK home office project license and in accordance with UK governmental regulations (Animal Scientific Procedures Act 1986).

### 3.5. Intranasal HSV-2 Challenge Model

Groups of eight to ten mice were lightly anesthetized under general anesthesia Isoflurane and 50 µl of 10^5^ or 10^6 ^pfu HSV-2 strain 333 was slowly pipeted onto the tip of the nose. The mice were kept on their backs until complete recovery from anesthesia. Following infection mice were weighed and scored daily (by operators who were blinded as to the identity of the treatment groups) using the following clinical scores: 0 = no apparent infection, 1 = piloerection and/or breathing difficulties, 2 = reduced movement/dull squinting eyes, 3 = sores on eyes, ocular discharge and/or hunched, 4 = symptoms above 3, 5 = severe respiration difficulties, 6 = paralysis. Any mouse approaching a score of 5 was sacrificed.

### 3.6. Viral Plaque Assays

Vero cells were grown to confluence in 12 well plates. Samples were diluted in EMEM medium and added to the confluent monolayers followed by a one hour incubation period at 37 °C. The inoculum was then removed and 1 ml of overlay (4 % Carboxymethyl cellulose) solution was added and incubated for another 2 to 3 days at 37 °C. One hour prior to reading the plates, 1 mL of 2 % neutral red solution was added to enhance the visibility of the plaques.

### 3.7. ELISpot

Splenocytes from infected mice were harvested by mechanical disruption and washed twice in serum free cell culture media. The splenocytes were then resuspended in R10 and plated out on IFN-γ (7.5 µg/mL, AN18 or 1-DK1 diluted in PBS with 0.5% FCS), (Mabtech, Sweden), coated PVDF (polyvinylidene difluoride)-backed microplate (Millipore), at a concentration of 5 × 10^5^ cells/well. Prior to the addition of the splenocytes, the PVDF plates were washed and blocked with 10% FCS (Sigma) for 4 h at 37 °C. Individual HSV-2 9mer peptides at a final concentration of 20 µg/mL were then added to the wells and cultured for 18 hr at 37 °C, in a humid 5% CO_2_ incubator. The next day the plate was developed using the manufacturer’s instructions (Mabtech, Sweden). Spots were read and counted on an automated ELISpot counter (AID Diagnostika GmbH). Results are expressed in Spot Forming Cells (SFC) per 5 × 10^5^ cells with standard error means (SEM). Responses equal to or greater than 90 SFC per million splenocytes were considered positive responses. 

### 3.8. Cell Lines

T2 (174 × CEM.T2) mutant hybrid cell line derived from the T-lymphoblast cell line CEM was obtained from the ATCC. T2 cells lack the functional transporter associated with antigen processing (TAP) heterodimer and fail to express normal amounts of HLA-A*0201 on the cell surface. HLA-A*0201 surface expression can be stabilized with exogenous loading of peptide able to bind to the MHC class I molecule. The T2 cell line was maintained in RPMI 1640 (Sigma-Aldrich) supplemented with 10% heat-inactivated fetal calf serum (FCS) and 100 U of penicillin/mL, 100 U of streptomycin/ml (collectively called R10) (Sigma-Aldrich).

### 3.9. Detection of CD107a Mobilization in T cells

CD8+ T cell degranulation has been shown to be a prerequisite for perforin and granzyme mediated cell lysis [43]. Degranulation can be measured by the mobilization of CD107a/LAMP-1 from the lysosomal membrane compartment to the cell surface which results in the increase of CD107a cell surface expression [44]. Briefly, 2 × 10^6^ of mixed splenocytes were transferred into 96-well V bottom FACS plates (BD) in R10 media medium and stimulated with peptide (2 µg/mL) in the presence of anti-CD107a-FITC (BD Pharmingen) (1 µg/well) for 4 hours. ConA (10µg/mL) (Sigma) and irrelevant peptide were used as positive and negative controls, respectively. Cells were then washed and stained with anti-CD8-APC and anti-CD4-PE antibodies (BD Pharmingen). The cells were washed and fixed using cytofix solution (BD Biosciences) according to manufacturer’s instructions. A minimum of 1 × 10^6^ cells were acquired on an FACS canto II using a 96well HTS module (BD Biosciences). Analysis was performed using FACS Diva. Frequencies are presented as a percentage of CD107a positive cells from total CD8+ cells with the irrelevant control frequencies subtracted.

### 3.10. Peptide-induced T2 stabilization assay

T2 cells were cultured in R10 media in 5% CO_2_ at 37 °C. Peptides from ICP27, VP22 and VP13/13 were tested for their ability to bind to and stabilize HLA-A*02 molecules on the surface of T2 cells. T2 cells (2 × 10^5^) were suspended in R10 media and distributed into 96-well plates (BD). The cells were incubated with the peptides at 50 µg/mL final concentration. The media was also supplemented with ß_2_-microglobulin (Sigma) at 2.5 µg/mL for 18 h at 37 °C. The T2 cells were then stained with anti-HLA-A*02-FITC BB7.2 monoclonal antibody (BD Pharmingen) and analyzed after 20 minutes on ice. The number of stabilized HLA-A*02 molecules on the surface of T2 cells was determined by fluorescent intensity. The ability of the peptide to combine with the HLA-A*02 was calculated as follows: AI = (MFI_1_ – MFI_0_)/(MFI_2_ – MFI_0_), where AI is the affinity index for HLA-A*02, MFI_1_ is the mean fluorescence index (MFI) of staining T2 cells treated with the peptide, MFI_2_ is the MFI of staining T2 cells without peptide treatment, and MFI_0_ is the MFI of T2 cell staining with an isotype control antibody. AI peptide scores equal to or greater than 1.5 displayed high affinity, 1.25-1.49 intermediate affinity and 1.1-1.25 were regarded as low affinity peptide as previously described [27]. Peptide specific T cells were generated from healthy donors using peptides that displayed intermediate and high affinity binding to T2 cells.

### 3.11. In vitro Peptide Specific T Cell Proliferation

To obtain peptide specific T cells, PBL were taken from healthy donors who were HLA-A*02 positive and processed as described previously [45]. PBL donor lymphocytes (1185) were pulsed every 10 days with 25 µg/mL of selected peptides in the presence of IMDM, 5% human serum (Sigma) and 200 units/mL IL-2 (R&D systems). Cells that responded to HSV-2 peptide were later maintained in 25 ng/ml IL-15 (R&D Systems, Minneapolis, MN) to allow low level proliferation and survival. 

### 3.12. Generation and Expansion of HSV-2 Peptide Specific T Cells from a Healthy HLA-A*02+ donor.

PBL were derived from a HLA-A*02+ donor and stimulated with peptides from ICP27_(314-322),_ ICP27_(428-436),_ VP22_(146-154),_ VP22_(173-181)_ and VP13/14_(242-150)_. Stimulations were performed with a one hour pulse of 20 µg/mL peptide and 25 ng IL-15 prior to growth for seven days. ELISpots were performed after each stimulation to monitor progress. After 4 stimulations cells were assessed for peptide specific responses by an overnight IFN-γ ELISpot assay. ELISpots were performed as described above, using Mabtech human IFN-γ ELISpot kit (Mabtech, Sweden).

### 3.13. DNA Vaccine Construction and Immunization

DNA vaccines were constructed and formulated onto gold particles for PMED delivery using methods previously described [46]. Briefly, codon optimized cDNA sequences of ICP27, VP22 and VP13/14 were synthesized (GeneArt AG, Regensburg, Germany) and subcloned into pPML7857, pPML7858 and pPML7871 plasmids respectively. Recombinant mRNA expression was confirmed for all vectors by RT-PCR following transfection of CHO cells. Monovalent DNA vaccines for each plasmid were precipitated onto gold beads ranging from 1–3 μm in diameter and filled into cassettes for helium accelerated DNA-gold particle delivery by the single use ND10 device [46]. Each actuation of the ND10 device was formulated to deliver 2 μg of DNA on 1 mg of gold. BALB/c mice (n = 6) (6–8 weeks old) were intradermally immunized with a single actuation from the ND10 device encoding the DNA constructs on gold [23]. All immunization procedures were carried out under the authority of a UK home office project license.

## 4. Conclusions

We have used linear based epitope detection methods to characterize several potential CD8+ T cell epitopes from HSV-2 proteins ICP27, VP22 and VP13/14 in an HSV-2 infection model in BALB/c mice. Experimental validation was performed using the HSV-2 infected mouse model and the results compared with responses elicited by DNA encoded vaccines. Responses were shown by ELISpot generally to be of a higher magnitude in the infection model than in vaccinated animals. These epitopes could be used to monitor peptide specific T cell responses after DNA vaccine delivery with the HSV-2 antigens ICP27, VP22 and VP13/14. It is possible that an optimized vaccination strategy may generate a sufficiently strong and broad response to mimic the responses seen in the infected animals. 
